# Exploring the anti-erosive potential of film-forming polymers and fluoride on salivary pellicle modification

**DOI:** 10.1007/s00784-025-06634-5

**Published:** 2025-11-01

**Authors:** Letícia Oba Sakae, Samira Helena Niemeyer, Alessandra Bühler Borges, Taís Scaramucci, Thiago Saads Carvalho

**Affiliations:** 1 Department of Restorative, Preventive and Pediatric Dentistry, University of Bern, Freiburgstrasse 3, Bern, CH-3010 Switzerland; 2 Department of Restorative Dentistry, School of Dentistry, University of São Paulo, Av. Prof. Lineu Prestes 2227, São Paulo, 05508-000 SP Brazil; 3 Department of Restorative Dentistry, Institute of Science and Technology, São Paulo State University, Av. Engenheiro Francisco Jose Longo, 777, São José dos Campos, São Paulo 12245-000 Brazil; 4 Department of Restorative, Preventive and Pediatric Dentistry, Dental Research Center, University of Bern, Freiburgstrasse 3, Bern, CH-3010 Switzerland

**Keywords:** Acquired salivary pellicle, Dental erosion, Enamel, Polymers, Sodium fluoride

## Abstract

**Objectives:**

To investigate the effect of solutions containing film-forming polymers on salivary pellicle modification for protection against dental erosion.

**Materials and methods:**

Three polymers were tested: Chitosan, Carbopol, and Linear sodium polyphosphate (LPP), each with or without sodium fluoride (F). Control groups were deionized water, F only, and tin/fluoride (Sn/F, positive control). Bovine enamel specimens underwent 10 cycles consisting of: 1 min salivary pellicle formation, 1 min modification with experimental solutions (*n* = 15/solution), 28 min salivary pellicle formation, and an erosive challenge (1 min for the first 5 cycles, 5 min for the last 5 cycles, totaling 30 min). Relative surface hardness (rSH), surface loss (SL), and calcium release (CaR) were analyzed after 5 and 30 min of erosion. The wettability of enamel in the presence and absence of the pellicle promoted by the solutions was also evaluated.

**Results:**

Chitosan showed a protective effect, with best results in rSH after 30 min in the no F subgroup, and when combined with F, it had similar performance to Sn/F. Carbopol showed the least protection, with the greatest loss of rSH and highest CaR, while LPP performed similarly to Carbopol whether combined or not to F. All polymer solutions exhibited higher wettability in the presence of the pellicle.

**Conclusions:**

Only Chitosan modified the pellicle and improved its protective effect against erosion. The pellicle may hinder the adsorption of negatively charged polymers, reducing their protective potential.

**Clinical Relevance:**

Chitosan may be a promissing agent for incorporation into erosion-preventive oral care products.

## Introduction

Frequent contact between non-bacterial acids of either extrinsic or intrinsic origin with the tooth surface can lead to dental erosion. This condition is characterized by irreversible mineral loss, resulting from a softened layer on the tooth surface, followed by cumulative loss of dental hard tissues. As the condition progresses, the underlying dentin may become exposed, leading to a negative impact on individuals’ quality of life due to dentin hypersensitivity [1]. Epidemiological studies highlight the high prevalence of dental erosion worldwide [2], with dentin exposure already being observed in children, adolescents, and young adults [3]. Consequently, dental erosion has become a growing concern in modern dentistry.

Given the severity of this condition, dentists and researchers have focused their efforts on developing effective preventive strategies. Among the most widely recommended options, topical fluoride application, especially when combined with stannous ions, is considered the gold standard for preventing dental erosion [4]. However, stannous formulations present some drawbacks, such as an unpleasant taste and the potential for tooth staining with prolonged use [5]. These limitations have led to the exploration of alternative or supplementary preventive strategies that might provide protection without the downsides associated with tin/fluoride.

Saliva plays a crucial role in the natural protection against dental erosion through the formation of the salivary pellicle, a thin layer formed by the adsorption of proteins, peptides, lipids, and other macromolecules onto the tooth surface [6]. The salivary pellicle protects the tooth surface from direct acid contact; however, as a semi-permeable network, its protection is limited when faced with frequent erosive challenges [7]. In an attempt to compensate for this limited protection, recent research has focused on enhancing the pellicle’s protective properties by modifying its composition [8–11]. Some studies have demonstrated promising results in obtaining more acid-resistant salivary pellicles using a variety of proteins [11–13] or polyphenols [8, 10] to modify the pellicle. Thus, understanding its interaction with different agents may be a promising and important field for further exploration.

Film-forming polymers, commonly employed in the food and cosmetic industries as thickeners and emulsifiers, have some anti-erosive properties [14–17]. These polymers can interact with the tooth surface, forming a protective layer that reduces ionic exchanges between acids and the mineral content of the tooth [14]. In the presence of a mature salivary pellicle, however, previous in situ studies raised a hypothesis of a possible competition between the salivary proteins and the polymers for binding sites on the enamel surface [18, 19]. To possibly overcome this competition, we hypothesized that if the polymers were applied during the initial salivary pellicle formation, there would be more available sites on the enamel surface for the polymers to bind or more chances for the polymers to interact with the salivary proteins.

In a recent in vitro study, five film-forming polymers — tested at different concentrations and pH values — were screened for their ability to modify the salivary pellicle and reduce hydroxyapatite dissolution [20]. Among them, Carbopol, Chitosan, and Linear Sodium Polyphosphate (LPP), either in association with sodium fluoride or not, were the most promising. These three polymers possess different characteristics but share the potential to interact with the enamel, but the mentioned study was not fully representative of clinical conditions. Thus, the present in vitro study aimed to evaluate the impact of solutions containing Carbopol, Chitosan, or Linear Sodium Polyphosphate at optimal concentrations and pH levels [20] in the presence of an early-stage salivary pellicle. Additionally, the combination of these polymers with fluoride could further potentialize their protective effect, so the present study also tested these polymers with and without fluoride on modifying the salivary pellicle and protecting bovine enamel against dental erosion. To gain a better understanding of the underlying mechanisms, erosion was tested under both mild and more severe conditions, allowing for the evaluation of polymer and fluoride effects across different levels of erosive challenges. The interactions between the polymers and the salivary pellicle were further investigated by assessing the enamel wettability promoted by the solutions, both in the presence and absence of the pellicle.

## Materials and methods

This study followed a completely randomized in vitro model with two experimental factors: test solutions at 4 levels (deionized water, Chitosan, Carbopol and Linear Sodium Polyphosphate) and fluoride, at 2 levels (presence and absence) (*n* = 15). Specimens of bovine enamel underwent 10 erosive cycles: where in the first 5 cycles, the specimens were submitted to 1 min erosion each (totaling 5 min erosion) and then for the next 5 cycles, submitted to 5 min each (totaling 30 min erosion). The response variable evaluated was the relative surface hardness (rSH); total amount of calcium released in the citric acid (CaR); and surface loss (SL). Additionally, the ability of the experimental solutions to wet the enamel surface was characterized.

### Ethical aspects

The study was carried out in accordance with the approved guidelines and regulations of the Ethics Committee (Kantonale Ethikkommission: KEK). As the teeth and saliva were pooled and were categorized as “irreversibly anonymized” by the ethics committee, no previous approval was necessary.

### Specimen Preparation

For this study, 189 freshly extracted bovine incisors were obtained from young animals, with an average age of 3 years. The teeth were stored in a 0.1% thymol solution for disinfection and under refrigeration at 4 °C, until the time of their use. The crowns were separated from the roots using a low speed cutting machine (IsoMet Low Speed Saw, Buehler, Lake Bluff, USA), using continuous water cooling.

Enamel fragments 4 mm wide x 4 mm long were obtained from the central region of the buccal surface of the crowns, using a high-precision automatic cutting machine (IsoMet 1000, Buehler, Lake Bluff, USA), also using continuous water cooling. The specimens were embedded in acrylic resin (Paladur, Heraeus Kulzer GmbH, Hanau, Germany) and then, flattened with grinding papers of silicon carbide with decreasing grain (18.3 μm to 3 μm). This procedure was standardized to remove the most superficial 200 μm of enamel. After that, the specimens were stored in a mineral solution (1.5 mM CaCl_2_, 1.0 mM KH_2_PO_4_, 50 mM NaCl, pH = 7.0 [21]) until the beginning of the experiments, when they received the final polishing with 1 μm diamond paste (DP-Stick, Struers, Ballerup, Denmark), for 1 min, to remove the mineral deposits that might have formed during storage in the mineral solution. The flattening and polishing procedures of the specimens were carried out in a polishing machine (TegraPol-15, Struers, Ballerup, Denmark), using continuous water cooling. After each grain size, the specimens were sonicated in deionized water.

A total of 135 specimens were used for the cycling while 54 specimens were used to characterize the wettability of enamel surfaces by the experimental solutions. The specimens that were used for the cycling had half of their surface covered by an unplasticized polyvinyl chloride (UPVC) adhesive tape to protect the reference area, while the other half was left exposed to the subsequent treatments.

### Collection of stimulated human saliva

Stimulated human saliva was obtained from 18 healthy donors, aged 20–61 years and from both genders. The donors presented good general health (without active caries, periodontal disease, erosive tooth wear, with normal salivary flow, and not using long-term medications that could interfere in the salivary flow). They were informed not to eat or drink (except water) for 1 h before the saliva collection, which was performed in the morning. The donors chewed on a paraffin wax for 10 min, and all the stimulated saliva was collected in chilled vials. After the collection, the saliva was pooled and centrifuged for 20 min at 4 °C (4000 *g*). The supernatant was collected and then, protease inhibitors (Phenylmethane sulfonyl fluoride, N-Ethylmaleimide, and Phenanthroline [Sigma-Aldrich, St Louis, USA]) were added to the saliva in a volume ratio of 1:100 of inhibitor for saliva, respectively [22]. Right after, the saliva was divided in small aliquots and stored at −80 °C until use.

### Experimental groups

There was a total of 8 solutions at two factors: test substance and presence/absence of fluoride (Table 1). An additional tin/fluoride solution was the positive control (Sn/F, sodium fluoride + stannous chloride, Sigma Aldrich, St Louis, USA), containing 225 ppm F^−^ and 800 ppm Sn^2+^ [23]. Three polymers were tested in this study: Chitosan (Chitosan 80/500; molecular weight ~ 350 kDa; degree of deacetylation 80%; viscosity 500 mPa·s, Heppe Medical Chitosan GmbH, Halle, Germany), Carbopol (Polyacrylic acid, Carbopol 980; molecular weight ~ 0.1 kDa, Lubrizol Inc., Cleveland, USA), and LPP (Linear Sodium Polyphosphate; molecular weight ~ 2.5 kDa, linear chain, average chain length of 24 phosphate units, Merck Millipore, Darmstadt, Germany). Solutions of each one of these polymers, alone and in association with sodium fluoride (F), were prepared fresh prior the beginning of the experiment. For sodium fluoride solutions (NaF, Merck, Darmstadt, Germany), the concentration usually found in mouth rinses was chosen (225 ppm F^−^ or approximately 0.5 g/l) [24]. The experimental groups, concentrations and pH are described in Table 1.

**Table 1:** Experimental groups

Group	Test substance	Fluoride content NaF	Main active ingredient (g/l)	pH
Negative control	Deionized water	0	-	5.7
F	NaF	225 ppm	-	4.5^1^
Chitosan	Chitosan	0	5	4.5^1^
Chitosan + F	Chitosan + NaF	225 ppm	5	4.5^1^
Carbopol	Carbopol	0	1	4.5^1^
Carbopol + F	Carbopol + NaF	225 ppm	1	3.942
LPP	LPP	0	5	5.25^2^
LPP + F	LPP + NaF	225 ppm	5	5.3^2^
Sn/F (positive control)	SnCl_2_ + NaF	225 ppm	1.277 (SnCl_2_)	4.5^1,3^

^1^ pH was adjusted to 4.5 with KOH 1 M or HCl concentrated solution

^2^ pH not adjusted, but left in the native state [20]

^3^ 2.3 g/l of gluconic acid sodium salt was added to this solution with stabilization purposes

### Categorizing the wettability of the enamel surface by the solutions

A drop shape contact angle device (Drop Shape Analysis System DSA 10 MK2, Krüss, Germany; needle Ø = 1.1 mm, drop volume 1 µl) was used for the wettability analysis of the enamel surface by the solutions, under two conditions: in the presence or absence of the salivary pellicle. For both conditions, 54 bovine enamel specimens were used, half of them were used without pellicle, while the other half was covered with the salivary pellicle for 1 min (37 °C, no agitation). Each experimental solution was then inserted into a syringe coupled to the device, and on each enamel specimen (*n* = 3 specimens with and *n* = 3 without pellicle for each solution), three droplets were placed on different locations. The average of the two contact angles from each droplet was calculated, totalizing 9 values per solution per condition. These values have an inverse relation with the wettability results, which means that smaller angles imply higher wettability, and vice-versa.

### Study design

The specimens underwent a total of 10 cycles: initially 5 cycles, consisting of a mild erosion (1 min erosive challenge), and subsequently 5 more cycles with a more severe erosion (5 min erosive challenge). All in all, the experiment consisted of a total of 30 min erosion after completion of the 10 cycles.

For the cycles, an aliquot of freshly thawed human saliva was placed on each specimen for 1 min (37 °C, no agitation) to allow an initial salivary pellicle formation. Then, the specimens were immersed with one of the experimental solutions for 1 min (25 °C, 70 rpm, travel path 50 mm), followed by another 28 min of salivary pellicle formation, for the modification of the pellicle. Finally, the specimens were submitted to an erosive challenge, each in an individual vial, with 1% citric acid (pH = 3.6) (25 °C, 70 rpm, travel path 50 mm) [25]. After each of these procedures, the specimens were washed with deionized water and dried with oil-free air.

This sequence was repeated 10 times. For the first 5 cycles, the erosion time was 1 min each, totalizing 5 min, simulating a mild erosion. For the other 5 cycles, the erosion time was 5 min each, totalizing 30 min, simulating a more severe erosion.

**Fig. 1:**
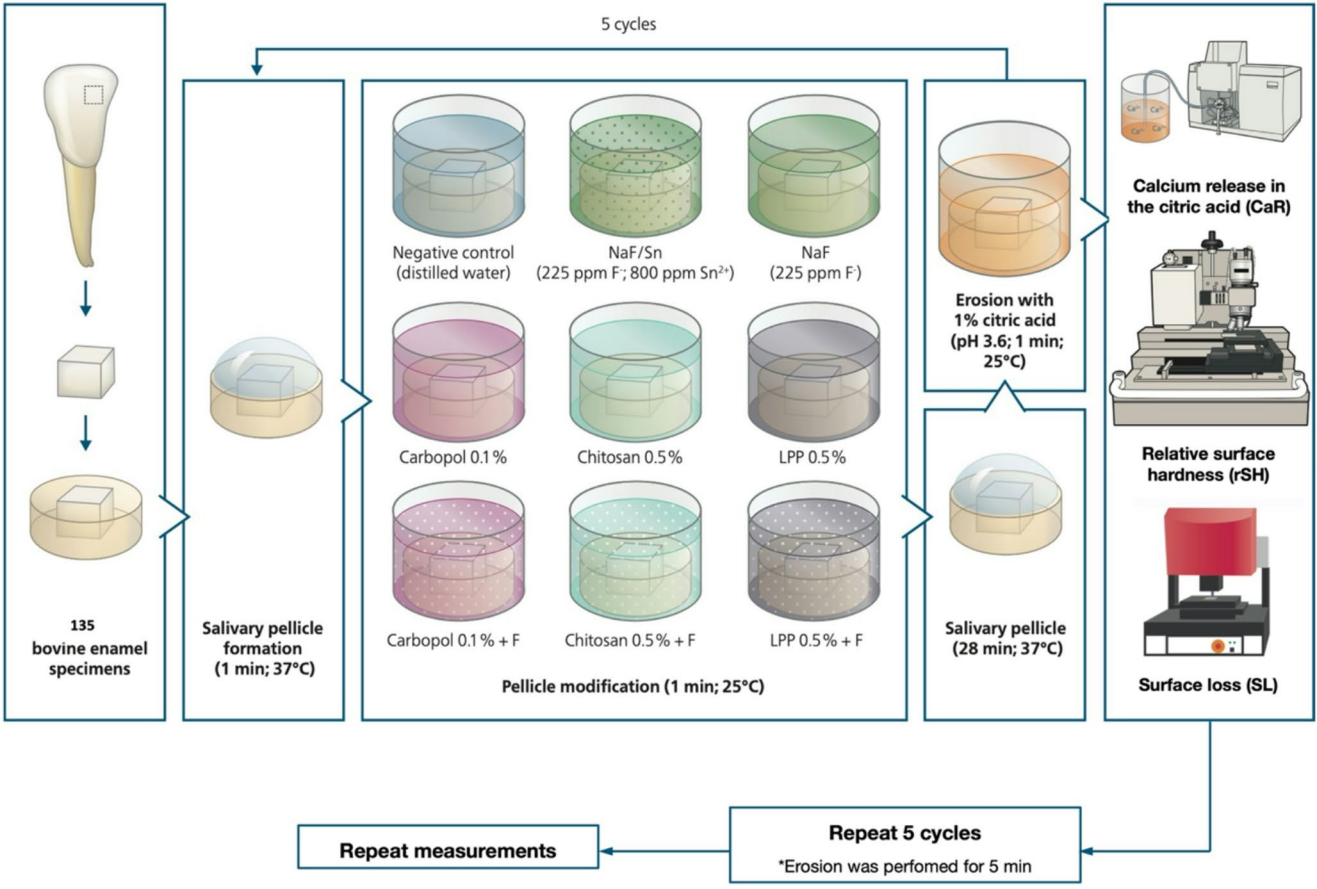
Experimental procedures

The response variables were: relative surface hardness (rSH), total amount of calcium released to the citric acid (CaR), and surface loss (SL). The sequence of the experimental procedures is illustrated in Fig. 1.

### Surface hardness measurement

Surface hardness (SH) was measured with a Vickers diamond, with 50.000 mN load and dwell time of 15 s (Fisherscope HM2000 XYp Nanoindenter, Helmut Fisher GmbH, Sindelfingen, Germany). For each SH measurement, six indentations were performed with 25 μm distance from each other. The average value from the six indentations was calculated and computed as the SH of each specimen. The SH was analyzed at baseline (SHi) and after 5 min (SHf_5_) and 30 min of erosion (SHf_30_). The relative SH (rSH) was calculated using the following formula: rSH = (SHf/SHi) x 100.

## Total amount of calcium released in the citric acid

After each cycle, two samples of 3 ml of citric acid were collected from each specimen’s individual vial, in which erosion was performed. At the end of the experimental procedures, the collected citric acid samples were mixed according to the specimen and total time of erosion (5 and 30 min). The amount of calcium released to the citric acid after 5 (CaR_5_) and 30 (CaR_30_) min, for each specimen, was determined using an atomic absorption spectrometer (AAnalyst 400, Perkin Elmer Analytical Instruments, Waltham, USA). Lanthanum nitrate (0.5% lanthanum nitrate hexahydrate: La[NO_3_]_3_.6H_2_O) was added to each mixture of citric acid, in order to eliminate the interference of other ions [26]. Then, the calcium concentrations were normalized to the treatment areas of the enamel specimens.

For this, the treatment area of each specimen was measured under a light microscope (Leica, M420) connected to a camera (Leica, DFC495), under 16x magnification. The software IM500 was used to contour the exposed area and calculate the exposed treated area from each specimen. The total amount of calcium released was expressed in nmol of Ca^2+^ per mm^2^ of enamel.

### Surface loss measurement

A central area of the enamel surfaces (2.5 × 1 mm) was scanned with an optical profilometer (MicroProf 100, FRT the art of metrology, Germany), for assessing the initial surface curvature using a specific software (FRT Mark III). Half of the specimen’s surface was covered with an unplasticized polyvinyl chloride (UPVC) adhesive tape, serving as a reference area. The other half was left uncovered to be exposed to the experimental procedures.

The same central area of the specimen surfaces was rescanned after 5 and then after 30 min of erosion. For measuring the surface loss, the mean height of the reference area was subtracted from the mean height of the test area. The initial values of each specimen were then subtracted from the final values, to counterbalance the initial surface curvature for each specimen.

### Statistical analysis

Data were evaluated with Shapiro-Wilk normality test and due to lack of normal distribution of some groups, non-parametric tests were used. Kruskal-Wallis for comparison between groups, considering each condition (presence or absence of F) separately, was performed for each response variable: rSH; total calcium released; and SL, after 5 and after 30 min of erosion. For wettability, the same test was conducted but considering each of the following conditions: presence or absence of the pellicle. Post hoc Dunn’s test with Bonferroni correction for multiple comparisons was performed. Then, to assess the effect of each solution in the presence and in the absence of fluoride; and in the presence and in the absence of the pellicle (for wettability only), each group was analyzed with Mann-Whitney U test comparing the conditions. The significance level was set at 5% and the analyses were carried out with IBM SPSS Statistics (IBM, Armonk, NY, USA).

## Results

### Wettability of the enamel surface by the solutions

**Fig. 2:**
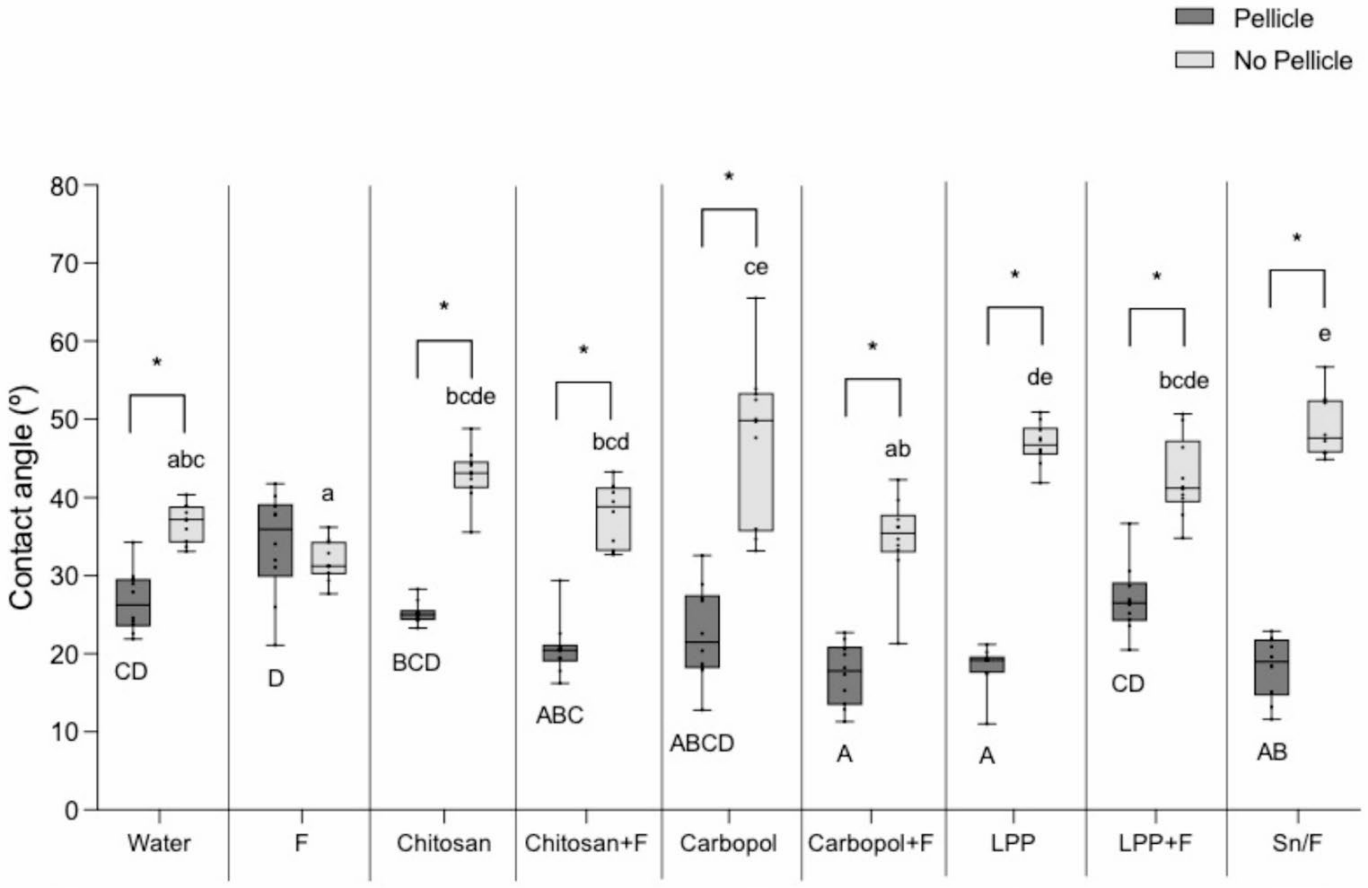
Contact angle between each solution drop and the enamel surface. Asterisks imply significant differences between the presence or absence of the pellicle among groups (*p* < 0.001). Different uppercase letters denote significant differences between groups in the presence of the pellicle (*p* < 0.05) and different lowercase letters indicate significant differences between groups in the absence of the pellicle (*p* < 0.05)

Figure 2 shows the contact angle for each solution and condition (in the presence or absence of the pellicle). In general, the presence of pellicle increased the wettability promoted by almost all solutions, except for F, which had similar wettability on both conditions.

In the presence of the pellicle, Sn/F, Carbopol + F and LPP promoted the highest wettability, significantly different from the negative control and F. By contrast, F resulted in the lowest wettability, not differing significantly from the negative control, LPP + F and Chitosan.

In the absence of the pellicle, Sn/F promoted the lowest wettability, while F promoted the highest wettability. All polymer solutions, except Carbopol + F and Chitosan + F, did not differ significantly from Sn/F. Carbopol + F did not differ significantly from F and the negative control. Chitosan + F did not differ significantly from the negative control.

### Relative surface hardness

**Fig. 3:**
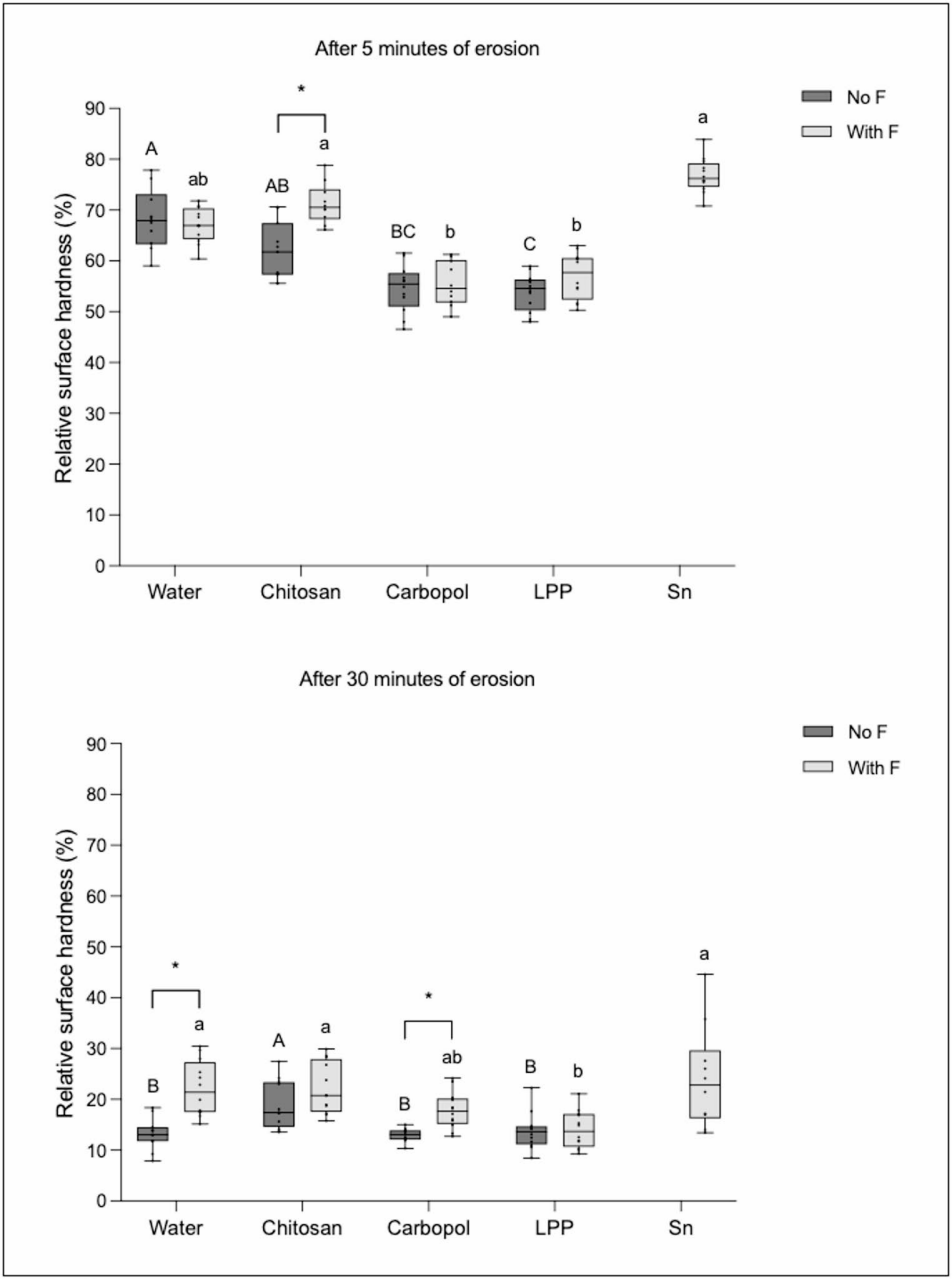
Relative surface hardness (%) after 5 and 30 min of erosion. Different lower-case letters denote significant diferences between the experimental groups in the presence of fluoride (with F). Different upper-case letters denote significant diferences between the experimental groups in the absence of fluoride (no F). Bar with asterisk shows significant difference between with F and no F within the same group

Figure 3 shows the results of relative surface hardness (rSH, %) after 5 and 30 min of erosion for both subgroups (with F and no F).

For the subgroup no F, after 5 min of erosion, the negative control and Chitosan showed the least erosion, differing significantly from LPP, which demonstrated the highest erosion. After 30 min of erosion, Chitosan showed the least erosion, differing significantly from the negative control, Carbopol and LPP.

For the subgroup with F, after 5 min of erosion, the positive control and Chitosan showed the least erosion, differing significantly from Carbopol and LPP. After 30 min of erosion, all solutions showed similar erosion, except LPP, which showed greatest erosion.

The presence of fluoride significantly reduced erosion for Chitosan after 5 min of erosion, while after 30 min, this effect was only observed for Carbopol and the negative control.

### Calcium released to the citric acid

**Fig. 4:**
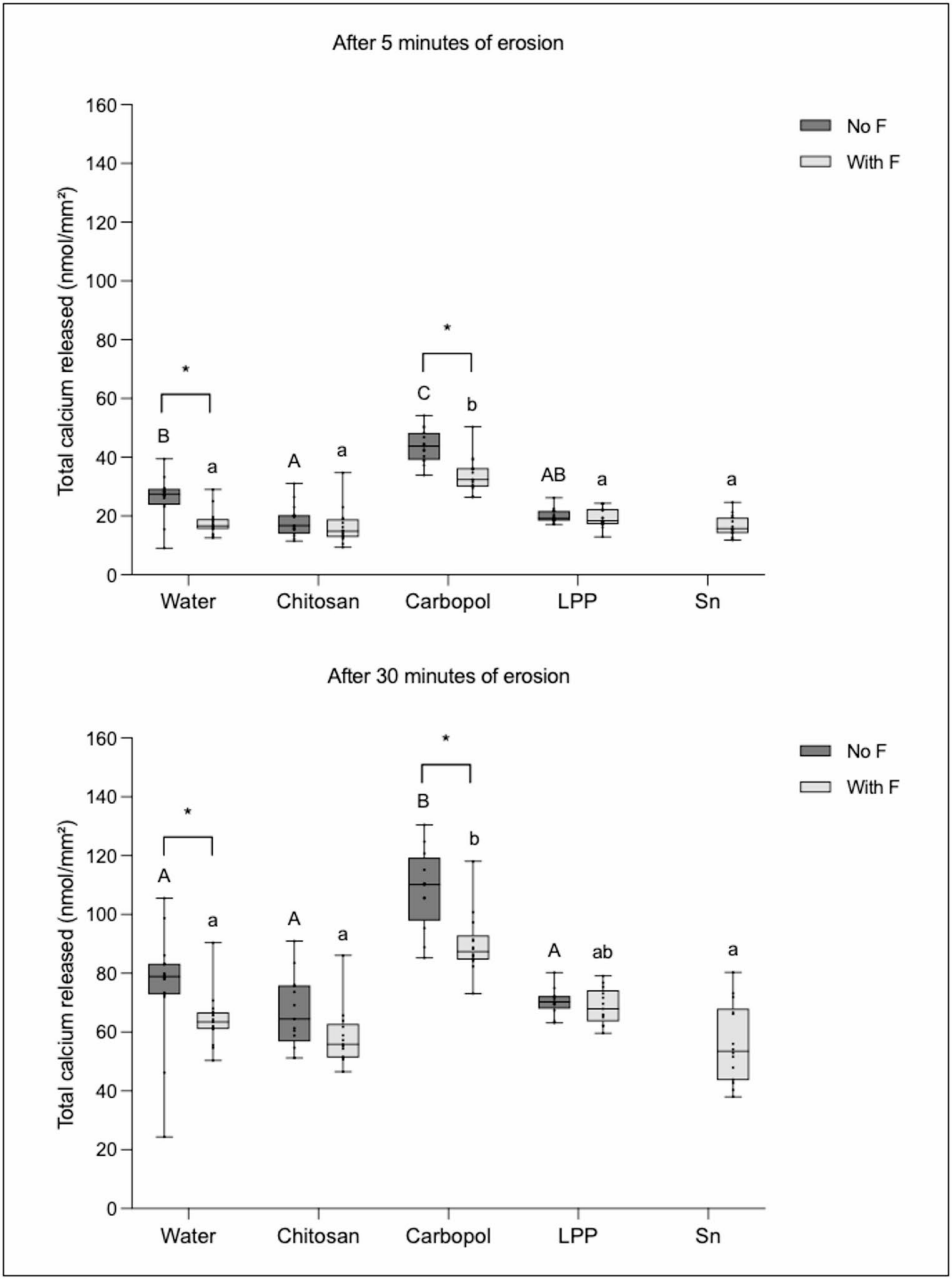
Total amount of calcium released to the citric acid after 5 and 30 min of erosion. Different lower-case letters denote significant diferences between the experimental groups in the presence of fluoride (with F). Different upper-case letters denote significant diferences between the experimental groups in the absence of fluoride (no F). Bar with asterisk shows significant difference between with F and no F within the same group

Figure 4 shows the results of total calcium released after 5 and 30 min of erosion for both subgroups (with F and no F).

For the subgroup no F, after 5 min of erosion, Chitosan showed the lowest values of calcium released, differing significantly from the negative control and Carbopol, which showed the highest values of calcium released. After 30 min of erosion, Chitosan, LPP and the negative control showed the lowest values of calcium released, differing significantly from Carbopol.

For the subgroup with F, after 5 min of erosion, Chitosan, LPP, NaF and the positive control showed the lowest values of calcium released, differing significantly from Carbopol. After 30 min of erosion, Chitosan, NaF and the positive control showed the lowest values of calcium released, differing significantly from Carbopol.

After 5 and 30 min of erosion, the presence of fluoride reduced significantly the calcium release for Carbopol and the negative control.

### Surface loss

**Fig. 5:**
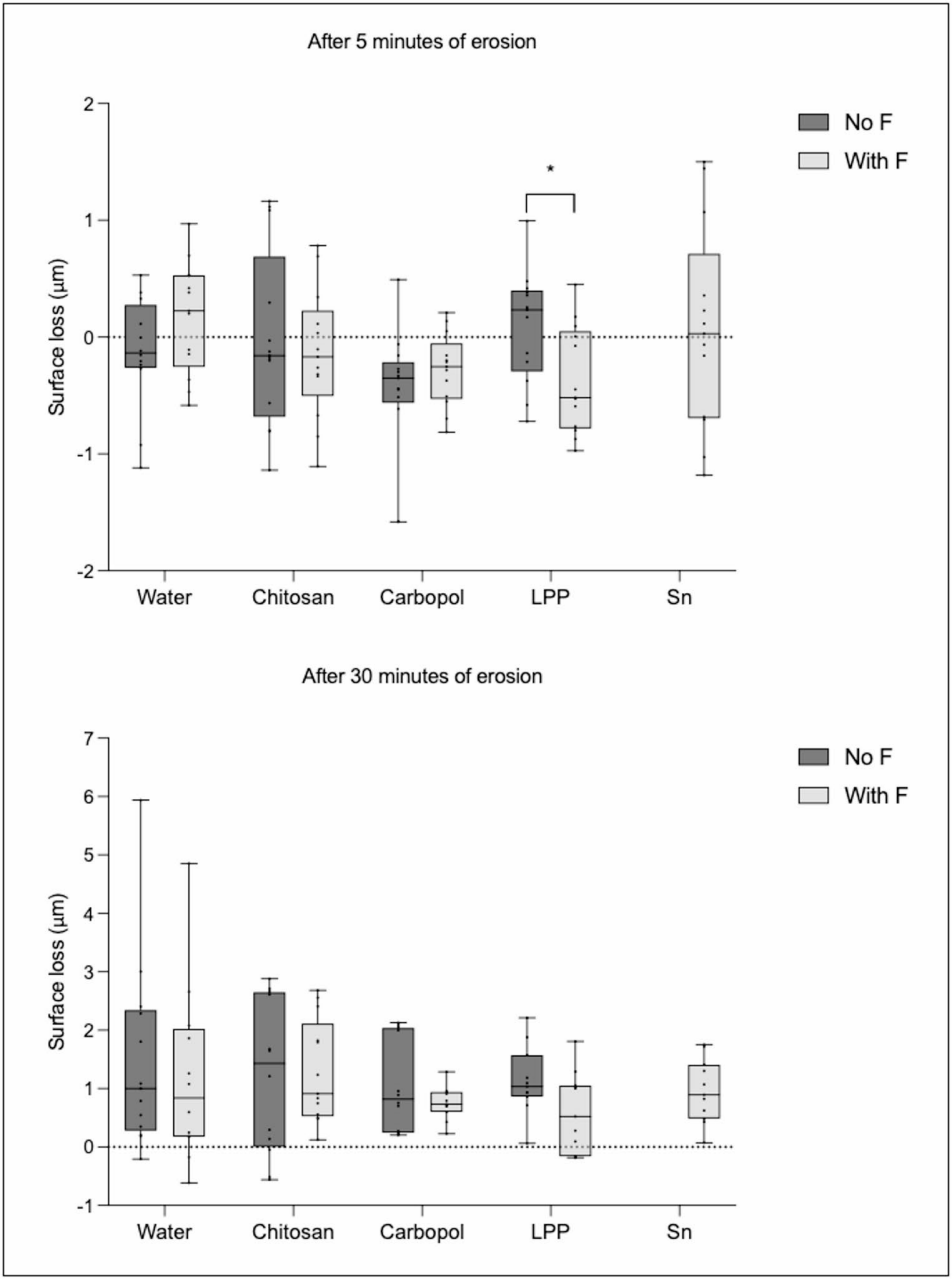
Surface loss after 5 and 30 min of erosion. There were no significant diferences between the experimental groups. Bar with asterisk shows significant difference between with F and no F within the same group. Values above 0 mean loss of surface; values bellow 0 mean gain in surface

Figure 5 shows the results of surface loss after 5 and 30 min of erosion for both subgroups (with F and no F). There were no significant diferences between the experimental groups, for both subgroups and periods of erosion. After 5 min of erosion, the presence of fluoride reduced significantly the surface loss for LPP.

## Discussion

This study is, to our knowledge, the first to systematically investigate pellicle modification with film-forming polymers as a preventive approach against dental erosion. Unlike previous studies, which typically applied polymers directly to enamel surfaces without a pellicle (using mineral solutions that simulate saliva in terms of mineral content but lack salivary proteins to form an actual pellicle) [14, 15], to in vitro pellicles formed without protease inhibitors (also lacking salivary proteins) [16] or to in situ pellicles formed after 2 h (representing a more mature pellicle) [18, 19, 27], the present work specifically targeted the early stages of pellicle formation, when only the basal layer is established. Because there is a potential competition between the polymers and salivary proteins for binding sites on the enamel surface, this early timing could be critical for allowing the polymers to interact directly with the enamel and influence subsequent pellicle development. By addressing this early window of pellicle formation, the present study provides new insights into whether, and which, film-forming polymers are capable of modifying the pellicle to enhance protection against erosive challenges.

The erosion process in the oral cavity occurs always in the presence of saliva, so any rigorous assessment of anti-erosive agents must account for the presence of a salivary pellicle, which will surely mediate any protective effects against acidic challenges. In the present study, we assessed this interaction by characterizing the wettability of the the enamel surface treated with the solutions under two conditions: in the presence or absence of the salivary pellicle. We observed that the contact angle promoted by all the solutions, except for sodium fluoride, notably decreased in the presence of the pellicle; suggesting that the pellicle significantly increases the wettability of the enamel. The solutions can then disperse more easily over the pellicle-covered enamel surface, but it might also hinder the chances for the polymer to interact with the enamel surface itself.

Our current study, therefore, clarifies the mechanism of action of the polymers, and explains why we had obtained promising results with the polymer-solutions under previous in vitro conditions without saliva [14, 15] but not in in situ conditions when saliva was present [18, 27]. So, our previous hypothesis seems to be right, that the salivary pellicle seems to hinder the polymer adsorption onto the enamel surface due to a competition between the salivary proteins and the polymers for binding sites.

One of the possible main differences between the cited in vitro studies and the present one was the quality and quantity of the salivary proteins present in the collected human saliva used in the studies. The past studies did not add protease inhibitors to prevent protein degradation like the present one did. There is no evidence about the proteomic differences between an in vitro pellicle with or without the inhibitors, however, the protection against enamel erosion of an in vitro pellicle with inhibitors was significantly higher than the one without inhibitors [28]. This might indicate that the in vitro pellicle without inhibitors has less proteins in its composition, and therefore, more available binding sites on the enamel surface where the polymers could adsorb. Likewise, the presence of the salivary pellicle can influence the polymer’s interaction with the enamel surface and ultimately on the polymer’s protective ability.

The ability of the polymers in modifying the salivary pellicle and its protective effect was compared with three different control solutions, in the present study: deionized water (negative control), tin/fluoride (positive control) and sodium fluoride. The polymer’s solutions were compared to the negative control, while the polymer’s solutions associated with sodium fluoride were compared to sodium fluoride and the positive control. Additionally, each solution without fluoride was compared to its respective solution with fluoride. In general, we observed a protective effect from fluoride in the subgroup comparisons. This effect seems to rely on the adsorption of fluoride on the tooth structure, which can protect the underlying tooth structure against erosive challenges [4]. Although sodium fluoride does not necessarily prevent pellicle dissolution or detachment after acid exposure, it may promote protein re-attachment on the eroded enamel surface, thereby contributing to protection against demineralization [25].

Interestingly, in the comparison between deionized water and sodium fluoride, no protective effect of sodium fluoride was observed after 5 min of erosion; however, this effect became evident after 30 min based on the relative surface hardness analysis. This finding suggests that fluoride may be less effective under milder erosive conditions—such as a 1-min erosive challenge—but exerts a more pronounced protective effect under more severe challenges, such as a 5-minute erosive challenge. Considering that, in the oral cavity, the erosive fluids will rarely be in contact with the teeth for 5 min, it is more likely that fluoride will have a limited effect in vivo [29]. Therefore, beyond elucidating the mechanism of action of film-forming polymers in relation to the salivary pellicle, our findings also suggest that the protective effect of sodium fluoride is influenced by the severity of the erosive challenge.

Surprisingly, the positive control solution demonstrated similar levels of protection as of the sodium fluoride solution, as evidenced by the relative surface hardness and calcium released to the citric acid analyses. When the solution containing fluoride and stannous ions acts directly on the enamel surface, it forms a protective metal-rich layer, which is more acid resistant. In addition, tin can be incorporated into the enamel structure, which in turn reduces its solubility [23, 30]. However, the presence of the salivary pellicle could hinder tin incorporation into the dental structure; therefore, tin/fluoride could show less protection in the presence of the salivary pellicle than when acting directly on the dental surface [31], which could justify our results.

Based on the relative surface hardness and calcium release analyses, the subgroup Chitosan without fluoride was able to improve the protection of the salivary pellicle in comparison to the negative control, while the subgroup with fluoride showed a similar protection to the positive control. Previous investigations have also shown the beneficial effects of Chitosan against dental erosion, which is a biopolymer derived from the deacetylation of chitin. It is a positively charged molecule [32], which can establish strong bonds between their amino groups and negative zeta potential surfaces, like enamel, resulting in the formation of an organic protective layer [33]. In the presence of saliva, this effect could even be potentialized, by the possibility of also binding to the salivary pellicle, which is also a negatively charged protein film [34]. As a result, an organic multilayer of chitosan and salivary proteins could build up and protect against acid attacks [35, 36]. It is important to point out that the binding of Chitosan to dental surfaces is pH-dependent: under acidic conditions, the amino groups are protonated (-NH₃⁺), promoting strong electrostatic interactions with negatively charged enamel or pellicle proteins [37]. As the pH increases, these groups become deprotonated, reducing the binding and, consequently, the stability of the Chitosan layer, which may explain its protective effect in our study [38].

Carbopol is a high molecular weight synthetic polymer based on modified cross-linked acrylic acids [36]. The acrylic acid backbone provides a naturally anionic characteristic to the polymer due to the presence of carboxyl groups, especially under neutral pH conditions, in which the polymer achieves its maximum viscosity [39]. This characteristic also makes Carbopol a calcium-binding polymer, and it has been suggested that it can adsorb onto enamel and form a polymer-rich film [38]. Since we aimed to work with solutions, we adjusted the pH to more acidic values to obtain a more fluid preparation. However, the Carbopol solution at acidic pH, associated or not to sodium fluoride, not only failed to protect enamel against erosion, according to the relative surface hardness analysis, but notably resulted in greater calcium release into the citric acid solution compared to the negative control. This may be attributed to the physicochemical properties of Carbopol at low pH, where protonation of its carboxyl groups reduces its negative charge [40]. In theory, the reduced charge could allow for slightly better interaction with the negatively charged pellicle through weak hydrogen bonding. Nevertheless, the reduced ionization at acidic pH likely limits calcium binding, leaving more calcium available for dissolution. Although viscosity was not quantitatively measured in this study, the Carbopol solution appeared visually more viscous than the sodium polyphosphate and control solutions. This higher apparent viscosity might have contributed to the observed outcome by allowing the acidic polymer solution to remain longer on the enamel surface, potentially enhancing the contact time with the acid and thereby increasing demineralization. These factors combined may explain the observed exacerbation of calcium release into the citric acid.

LPP is an inorganic, long and condensed linear phosphate polymer with P-O-P chains (approximately 25 units long) [41]. These phosphate groups also make LPP an anionic polymer. In the present study, the LPP solution at pH 5.2–5.3 did not improve enamel protection compared to water and, when combined with sodium fluoride, appeared less effective than fluoride alone, especially after 30 min of erosion, as demonstrated by the relative surface hardness analysis. This could be related to the highly anionic nature of polyphosphates at this pH [42], which may limit their ability to interact with the negatively charged salivary pellicle and might even disrupt its structure by competing for calcium ions. Furthermore, because polyphosphates are calcium-binding polymers [43], under acidic conditions (such as during an erosive challenge), they might sequester calcium released from the enamel surface or pellicle, potentially enhancing demineralization. When combined with fluoride, polyphosphates might also interfere with the formation or stability of CaF₂-like deposits by binding available calcium [44], thereby possibly reducing the effectiveness of fluoride protection. Together, these mechanisms might explain the observed lack of efficacy of LPP under the present experimental conditions.

Our results, once again, highlight the importance of the salivary pellicle in the erosion process. The pellicle itself can slow down the erosion process as it functions as a semipermeable network, preventing the direct contact of erosive acids with the tooth surfaces [7], but the protective effect of the pellicle is limited. Overtime, the acids can dissolve the pellicle more thoroughly and reach the enamel surface, leading to demineralization [7]. This was clearly portraited in the present study by comparing the salivary pellicle protection after 5 and after 30 min of erosion. After 5 min, where short (1 min) challenges were carried out, the pellicle could withstand the erosive challenges and protect the enamel. However, increasing the challenge time to 5 min (after 30 min of erosion), the protection given by the pellicle decreased significantly, as indicated by the relative surface hardness analysis. Still, surface loss was very low across all groups, with values generally ranging from less than 1 to 2 μm, including the negative control. Given that profilometric changes are only reliably detectable at depths of ≥ 1 μm [45], these results are very close to the detection threshold, which indicates minimal enamel wear under the experimental conditions. This suggests that the salivary pellicle itself may have contributed to limiting enamel erosion during the mild erosive challenges applied in this study.

In the present study, three complementary analyses were employed—relative surface hardness (rSH), calcium release, and surface loss—to evaluate the anti-erosive effects of the tested conditions. Each method provides information on different aspects of enamel erosion. rSH reflects early softening of the surface and therefore is particularly sensitive to initial changes in the superficial enamel layer. Calcium release indirectly quantifies erosion by measuring the amount of mineral lost to the citric acid [26]. Profilometry, in turn, detects physical loss of material but only above a certain detection threshold (approximately 1 μm) [45]. Consequently, small but biologically relevant changes may be observed in rSH and calcium release before they are detectable as surface loss. These differences in sensitivity and measurement principle may explain the apparent discrepancies between methods observed in the present study and highlight the importance of using complementary approaches to gain a more comprehensive understanding of erosive processes. However, the pellicle was not removed prior to the analyses, which could be considered as a limitation of our study. This was done in order to allow the evaluation of cumulative effects over different timepoints. Removing the pellicle after 5 cycles would have prevented comparison with the 10-cycles measurements, while removing it only after 10 cycles would not have provided a fair comparison with the 5-cycle results. Additionally, established protocols for complete pellicle removal require prolonged incubation and sonication in NaOCl [46], which could damage the eroded enamel surface and influence the results [47].

We therefore conclude that out of the three tested film-forming polymers, only Chitosan was able to modify the salivary pellicle and improve its protective effect against enamel erosion. Considering all the studies conducted so far, together with the outcomes of our present study, it seems that the negatively charged polymers (Carbopol and Linear Sodium Polyphosphate), might compete with the salivary proteins for binding sites on the enamel surface and have no interaction with the salivary pellicle. In addition, it seems that they have a better protective effect directly on the enamel surface, which potentially limits their clinical applications.

## Data Availability

All data generated or analyzed during this study are included in this article. Further enquiries can be directed to the corresponding author.
